# A Robust Oxygen Microbubble Radiosensitizer for Iodine‐125 Brachytherapy

**DOI:** 10.1002/advs.202002567

**Published:** 2021-02-10

**Authors:** Sheng Peng, Ruyuan Song, Qingguang Lin, Yanling Zhang, Yuanzhong Yang, Ma Luo, Zhihui Zhong, Xiaonan Xu, Ligong Lu, Shuhuai Yao, Fujun Zhang

**Affiliations:** ^1^ Department of Ultrasound Sun Yat‐sen University Cancer Center State Key Laboratory of Oncology in South China Collaborative Innovation Center for Cancer Medicine Guangzhou 510060 P. R. China; ^2^ Bioengineering Graduate Program Department of Chemical and Biological Engineering The Hong Kong University of Science and Technology Hong Kong 999077 P. R. China; ^3^ Department of Imaging and Interventional Radiology Sun Yat‐sen University Cancer Center State Key Laboratory of Oncology in South China Collaborative Innovation Center for Cancer Medicine Guangzhou 510060 P. R. China; ^4^ Department of Pathology Sun Yat‐sen University Cancer Center State Key Laboratory of Oncology in South China Collaborative Innovation Center for Cancer Medicine Guangzhou 510060 P. R. China; ^5^ Department of Mechanical and Aerospace Engineering The Hong Kong University of Science and Technology Hong Kong 999077 P. R. China; ^6^ Zhuhai Interventional Medical Center Zhuhai Precision Medical Center Zhuhai People's Hospital Zhuhai Hospital of Jinan University Zhuhai 519000 P. R. China

**Keywords:** brachytherapy, iodine‐125, oxygen microbubbles, tumor hypoxia, ultrasound

## Abstract

Iodine‐125 (^125^I) brachytherapy, a promising form of radiotherapy, is increasingly applied in the clinical treatment of a wide range of solid tumors. However, the extremely hypoxic microenvironment in solid tumors can cause hypoxia‐induced radioresistance to ^125^I brachytherapy, resulting in therapeutic inefficacy. In this study, the aim is to sensitize hypoxic areas in solid tumors using ultrasound‐activated oxygen microbubbles for ^125^I brachytherapy. A modified emulsion freeze‐drying method is developed to prepare microbubbles that can be lyophilized for storage and easily reconstituted in situ before administration. The filling gas of the microbubbles is modified by the addition of sulfur hexafluoride to oxygen such that the obtained O_2_/SF_6_ microbubbles (OS MBs) achieve a much longer half‐life (>3×) than that of oxygen microbubbles. The OS MBs are tested in nasopharyngeal carcinoma (CNE2) tumor‐bearing mice and oxygen delivery by the OS MBs induced by ultrasound irradiation relieve hypoxia instantly. The post‐treatment results of brachytherapy combined with the ultrasound‐triggered OS MBs show a greatly improved therapeutic efficacy compared with brachytherapy alone, illustrating ultrasound‐mediated oxygen delivery with the developed OS MBs as a promising strategy to improve the therapeutic outcome of ^125^I brachytherapy in hypoxic tumors.

## Graphical abstract

Tumor‐specific ultrasound‐activated oxygen microbubbles are developed to sensitize hypoxic tumor cells for ^125^I brachytherapy. The microbubbles, lyophilized for storage and easily reconstituted in situ before administration, have much better stability by the addition of sulfur hexafluoride into oxygen. Ultrasound‐mediated oxygen delivery with the microbubbles demonstrate a greatly improved therapeutic outcome of ^125^I brachytherapy in hypoxic tumors.

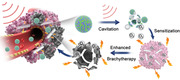

## 1 Introduction

Radiotherapy, which involves the use of high‐energy ionizing radiation (e.g., X‐rays) to induce DNA damage and further cause cellular necrosis via free‐radical oxygen species from the radiolysis of water, has been extensively applied in the clinical treatment of various types of cancers.^[^ ^1^ ^]^ However, normal cells are also killed nonspecifically in the path of external radiation beams intended to reach tumor tissues, leading to severe side effects.^[^ ^2^ ^]^ Furthermore, the outcome of radiotherapy is highly dependent on cell cycles,^[^ ^3^ ^]^ as tumor residuals may occur due to the low radiosensitivity of tumor cells in the G1/G2 phase during external radiotherapy.^[^ ^4^ ^]^ To suppress side effects and improve the therapeutic efficacy, ^125^I brachytherapy was developed to locally and continuously exert a high dose of radiation within tumor‐bearing regions by the direct implantation of ^125^I seeds into a tumor, where the radiation dose decreases rapidly with distance from the ^125^I seeds. Therefore, ^125^I brachytherapy has been increasingly accepted as a minimally invasive treatment for prostate cancer, lung cancer, brain cancer, etc.^[^ ^5^ ^]^

Apart from the side effects of radiation, the hypoxia within most solid tumors (at least 50–60%), typically arising from the abnormal vasculature and compromised diffusion in tumor microcirculation,^[^ ^6^ ^]^ is another critical issue jeopardizing the therapeutic outcome of radiotherapy. In a majority of solid tumors, the partial pressure of oxygen (pO_2_) is in the range of 2–18 mm Hg.^[^ ^7^ ^]^ Cellular radioresistance becomes pronounced at pO_2_ < 20 mm Hg^[^ ^8^ ^]^ since the presence of oxygen molecules is a prerequisite to stabilize the reactive oxygen species‐mediated DNA damage in order to further break the double‐stranded DNA, which would otherwise be restored by cellular thiol‐containing compounds.^[^ ^9^ ^]^ Hence, electron‐affinic chemicals (e.g., misonidazole and nitroimidazoles) that can react with DNA‐based radicals to permanentize DNA damage have been developed as radiosensitizers for hypoxic tumor cells.^[^ ^10^ ^]^ However, the promising efficacy of nitroimidazole and its derivatives has not been proven in clinical trials.^[^ ^11^ ^]^ Alternatively, chemotherapeutics such as paclitaxel and cisplatin are most commonly used as sensitizers in clinical practice.^[^ ^12^ ^]^ However, such concurrent radiochemotherapy exposes patients to a high risk of the additional severe side effects of chemotherapeutics, which may be overwhelming. Consequently, several strategies with minimal side effects have been proposed to address hypoxia‐induced radioresistance by directly delivering exogenous oxygen to tumor tissues via methods such as hyperbaric oxygen therapy,^[^ ^13^ ^]^ modified hemoglobin,^[^ ^14^ ^]^ and perfluorocarbon (PFC) Nano emulsions.^[^ ^15^ ^]^ However, oxygen delivery methods with hyperbaric oxygen therapy and modified hemoglobin still rely on the respiratory system to release oxygen into circulation; thus, the oxygen delivery efficiency of these methods is compromised by the irregular microcirculation and deteriorated diffusion in the tumor tissues. By leveraging the high oxygen solubility of PFCs (i.e., 40–50% v/v), ^[^ ^16^ ^]^ oxygen‐saturated PFCs can release oxygen in the hypoxic region through passive diffusion to sensitize hypoxic cells.^[^ ^17^ ^]^ The employment of PFC Nano emulsions incorporated with X‐ray absorbers (e.g., Bi_2_Se_3_ nanoparticles^[^ ^18^ ^]^ and TaOx nanoparticles^[^ ^19^ ^]^) has resulted in notably improved radiotherapy efficacy. Flu sol‐DA 20% (perfluorodecalin with perfluorotrypropylamine) has been studied clinically as a radiosensitizer specifically for glioblastoma.^[^ ^20^ ^]^ However, PFCs require high doses to achieve their high efficacy, which inherently compromises safety.

Alternatively, the use of oxygen microbubbles (MBs) seems to be a more clinically applicable method to address hypoxia‐induced radioresistance. First, the safety of MBs has been well proven by the use of phospholipids and albumin‐stabilized MBs filled with perfluoropropane or sulfur hexafluoride under different brand names (e.g., SonoVue, Definity, Optison, etc.) as ultrasound contrast agents (UCAs) in clinical practice for decades.^[^ ^21^ ^]^ With oxygen as the filling gas, lipid‐coated oxygen microbubbles (LOMs) have emerged as a promising new dual agent, simultaneously acting as UCAs and oxygen carriers.^[^ ^22^ ^]^ Second, LOMs carry substantially large volume fractions of oxygen (i.e., >80% v/v), which remarkably enhance the delivery efficiency compared with other oxygen delivery systems.^[^ ^23^ ^]^ In addition, the acoustic responsivity of LOMs enables local and transient oxygen release by ultrasound to relieve hypoxia in tumor tissues to a significant degree, thus enhancing the outcomes of oxygen‐dependent therapy modules such as sonodynamic therapy and radiotherapy.^[^ ^24^ ^]^ However, regardless of the improved safety and effectiveness of LOMs, several challenges remain for their clinical transition. First, LOMs are susceptible to prematurely releasing oxygen into the blood stream upon intravenous injection due to the high oxygen diffusivity and solubility.^[^ ^25^ ^]^ Second, LOMs suffer from gas dissolution, coalescence, and Ostwald ripening during storage, leading to marked product loss and a shift in MB size distribution.^[^ ^26^ ^]^ Therefore, oxygen MBs with good stability, easy shelf storage, appropriate size distributions, and high oxygen carrying capacities are highly desirable for the sensitization of hypoxic tumors to radiotherapy, especially for future clinical applications.

**Figure 1:**
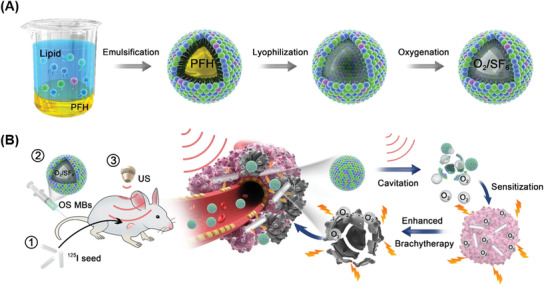
A) Schematic representation of the emulsion freeze‐drying strategy employed to fabricate the microbubbles. First, perfluoroheptane (PFH) is homogenized in a lipid solution to yield stable PFH emulsions. Second, freeze‐drying removes water and the sacrificial PFH cores to obtain hollow lipid microcapsules with polyethylene glycol as a cryoprotectant. Finally, the infusion of oxygen (O_2_) and sulfur hexafluoride (SF_6_) yields a gas‐filled lyophilisate matrix ready for O_2_/SF_6_ microbubble (OS MB) reconstitution. B) Illustration of the radiosensitizing effect of the OS MBs for cancer treatment with ^125^I brachytherapy via (1) ^125^I seed implant, (2) administration of the OS MBs, and (3) ultrasound‐triggered destruction of the OS MBs.

In this study, we present a new type of oxygen microbubble made by an emulsion freeze‐drying method. **Figure** **1**A illustrates the three key fabrication steps. First, perfluoroheptane (PFH) emulsions are formed by the homogenization of PFH in a lipid solution. Second, water and PFH are removed by lyophilization with polyethylene glycol as a cryoprotectant to obtain hollow lipid microcapsules. Third, the infusion of an oxygen and sulfur hexafluoride gas mixture yields a gas‐filled lyophilisate matrix. The resulting oxygen/sulfur hexafluoride (OS) MBs are ready for easy storage and reconstitution in situ prior to use. The addition of sulfur hexafluoride to oxygen as the filling gas of the OS MBs overcomes the poor stability of oxygen MBs alone. A series of in vitro and in vivo experiments were conducted to examine the stability, oxygen release kinetics, and ultrasound‐triggered destruction of the OS MBs for transient oxygen release. Figure 1B shows the strategy of using the OS MBs as a radiosensitizer for the ^125^I brachytherapy of hypoxic tumors. The therapeutic outcome of ^125^I brachytherapy combined with the ultrasound‐mediated OS MBs was evaluated in tumor‐bearing mice by comparing the tumor sizes and stained tumor slices after a treatment period of twelve days.

## 2 Results and Discussion

### 2.1 Design, Synthesis, and Characterization of OS MBs

We fabricated MBs with lipid shells following the procedure described in Figure 1A. Distearoyl phosphocholine (DSPC) was chosen as the main component of the shell because it provides an optimal tradeoff between in‐plane rigidity to stabilize the MBs and a relatively high gas permeation resistance.^[^ ^27^ ^]^ A small amount of palmitic acid (∼10%) was also incorporated into the outer shell to further increase the rigidity. However, the lipid monolayer is still permeable to oxygen and other gases. Oxygen with its high diffusivity (3.95 × 10^−9^ m^2^ s^−1^) and aqueous solubility (Bunsen coefficient of 0.0284) encapsulated in MBs continuously diffuses into surrounding aqueous environments, which can lead to the premature dissolution of oxygen MBs.^[^ ^28^ ^]^ Sulfur hexafluoride (SF_6_), the filling gas of commercial UCAs (i.e., SonoVue) with its much lower diffusivity (1.05 × 10^−9^ m^2^ s^−1^) and aqueous solubility (Bunsen coefficient of 0.005) was introduced into the filling gas to slow the gas dissolution.^[^ ^29^ ^]^ To prove this stabilization strategy, we fabricated three types of MBs for comparison: OX MBs (filled with oxygen), OS MBs (filled with O_2_ and SF_6_ at volume ratios of 9:1, 8:2, and 6:4, denoted as OS_9:1_ MBs, OS_8:2_ MBs, and OS_6:4_ MBs, respectively), and SF MBs (filled with SF_6_).

**Figure 2:**
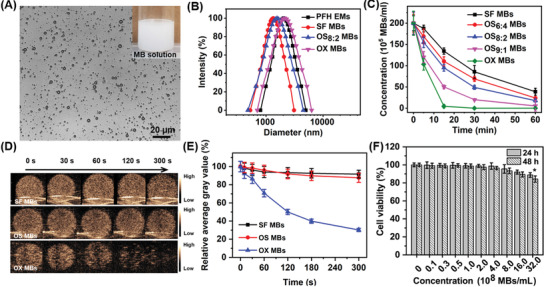
Characterization of microbubbles in terms of their morphology, size, biocompatibility, stability, and ultrasound contrast enhancement capacity. A) Optical microscopy images of OS MBs (filling gas: O_2_/SF_6_ = 8/2) reconstituted from lyophilized powders of the precursor PFH emulsions; the inset is an optical image of an OS MB solution in a glass vial. B) Size and size distribution of precursor PFH emulsions, SF MBs, OS MBs (filling gas: O_2_/SF_6_ = 8/2), and OX MBs. C) Concentration variations of SF MBs, OS_4:6_ MBs, OS_8:2_ MBs, OS_9:1_ MBs, and OX MBs incubated at 37 °C for 1 h (initial concentration: ∼2 × 10^7^ MBs mL^−1^). D) Ultrasound images of SF MBs, OS MBs, and OX MBs in the gel phantom at specific time intervals (MI = 0.06, frequency = 9 MHz). E) Relative average gray values of ultrasound images of SF MBs, OS MBs, and OX MBs to initial average gray values (*t* = 0 s) obtained from the time‐series ultrasound images in panel D. F) Cell viability of CNE2 cells treated with OS MBs at different concentrations for 24 and 48 h (*n* = 3, *p = 0.032 for 24 h and *p = 0.025 for 48 h compared with the control group).

**Figure** **2**A shows the spherical morphology of the OS_8:2_ MBs reconstituted by gently shaking the lyophilisate in 4 mL of PBS buffer, and the inset shows the stable milky MB solution in a glass vial. Figure 2B shows the size and size distribution of the PFH emulsions and the reconstituted MBs with different filling gases. Their main physicochemical characteristics, summarized in**1** showed that the components of the filling gases had a large impact on the size, size distribution, and concentrations of the reconstituted MBs. The OX MBs had the largest size and size distribution, while those of the SF MBs were the lowest among the three microbubble types. The diameters of the OS MBs with different O_2_/SF_6_ filling gas ratios were 1.63–1.94 µm and decreased with increasing SF_6_ proportion. The accuracy of the size measurements using dynamic light scattering might be compromised by the buoyancy force of the MBs. The production rate, defined as the ratio of the number of MBs reconstituted from the lyophilisates to the number in the PFH emulsions before lyophilization, was ∼0.10 for the OX MBs, ∼0.15 to ∼0.28 for the OS MBs, and ∼0.31 for the SF MBs. Compared with oxygen alone as the filling gas, a greater amount of SF_6_ in the filling gas led to a higher MB production rate, which may be attributable to the water‐insoluble SF_6_ suppressing bubble coalescence and dissolution during the reconstitution process. Although the production rate of the OS MBs was higher with more SF_6_ in the filling gas, the oxygen carrying capacity also decreased. The oxygen carrying capacity per vial of OS MBs was highest with O_2_/SF_6_ at a ratio of 8/2. To further evaluate the stability of the MBs, we monitored the concentration variations of the OX MBs, SF MBs, and three kinds of OS MBs incubated at 37 °C for 1 h. Figure 2C shows that the OS MBs were more stable than the OX MBs when the O_2_/SF_6_ ratio was 8/2 and 6/4. The half‐life of the OX MBs in terms of concentration was only ∼5 min, whereas the half‐lives of the OS_8:2_ MBs and OS_6:4_ MBs were similar (∼15 and ∼18 min, respectively). Considering both stability and oxygen loading capacity, we selected the OS_8:2_ MBs for further studies.

**Table 1:** Physical characteristics of precursor PFH emulsions, SF MBs (filling gas: SF_6_), OS_6:4_ MBs (filling gas: O_2_/SF_6_ = 6/4), OS_8:24_ MBs (filling gas: O_2_/SF_6_ = 8/2), OS_9:1_ MBs (filling gas: O_2_/SF_6_ = 9/1), and OX MBs (filling gas: O_2_). All microbubble solutions were reconstituted from the lyophilized powders of precursor PFH emulsion solutions at the same amount

	Size [µm]	PDI	Concentration [× 10^8^ MBs mL^−1^]
PFH emulsion	2.05 ± 0.15	0.20 ± 0.08	7.52 ± 0.33
SF MBs	1.59 ± 0.20	0.23 ± 0.05	2.29 ± 0.20
OS_6:4_ MBs	1.63 ± 0.25	0.27 ± 0.06	2.11 ± 0.23
OS_8:2_ MBs	1.75 ± 0.44	0.30 ± 0.05	1.96 ± 0.22
OS_9:1_ MBs	2.05 ± 0.38	0.34 ± 0.07	1.12 ± 0.15
OX MBs	2.12 ± 0.24	0.35 ± 0.06	0.73 ± 0.10

To assess the ultrasound contrast enhancement of the OS MBs and the volume of gas remaining in the MBs over time, the MBs were used as contrast agents for ultrasound imaging. Ultrasound images at specific time points were acquired at a low mechanical index (MI) to reduce the effect of ultrasound on gas dissolution. Initially, all three types of MBs exhibited strong ultrasound contrast enhancement. Subsequently, the ultrasound signal of the OX MBs decayed sharply, while that of the SF MBs and OS MBs remained relatively stable (Figure 2D). The ultrasound signal intensity of the OX MBs plummeted by ∼70% of its initial intensity after 5 min, whereas the ultrasound signal intensity of the OS MBs declined only slightly by ∼13% of its initial value (Figure 2E). The fast oxygen dissolution of the OX MBs accounts for the precipitous decrease in their ultrasound signal intensity, which was also revealed by the decrease in concentration of the OX MBs incubated at 37 °C without ultrasound exposure. In comparison, the presence of SF_6_ in the core of the OS MBs may significantly contribute to their greatly improved stability. A similar phenomenon has also been observed in MBs filled with a mixed gas of nitrogen and perfluorohexane vapors (∼15% v/v), which were more stable than their counterparts filled with nitrogen alone.^[^ ^30^ ^]^ In addition, F‐compound molecules (e.g., SF_6_ and C_6_F_14_) in the filling gas of MBs adsorbed on a lipid film, which served as a co‐surfactant, were shown to be favorable for lowering the surface tension and Laplace pressure of MBs, which further improved the stability.^[^ ^31^ ^]^ In addition, a biosafety evaluation revealed that the OS MBs possessed excellent biocompatibility at concentrations as high as ∼3.2 × 10^9^ MBs mL^−1^, as shown in Figure 2F.

### 2.2 Acoustic Oxygen Release Behavior and Oxygenation of Hypoxic Tumor with OS MBs

**Figure 3:**
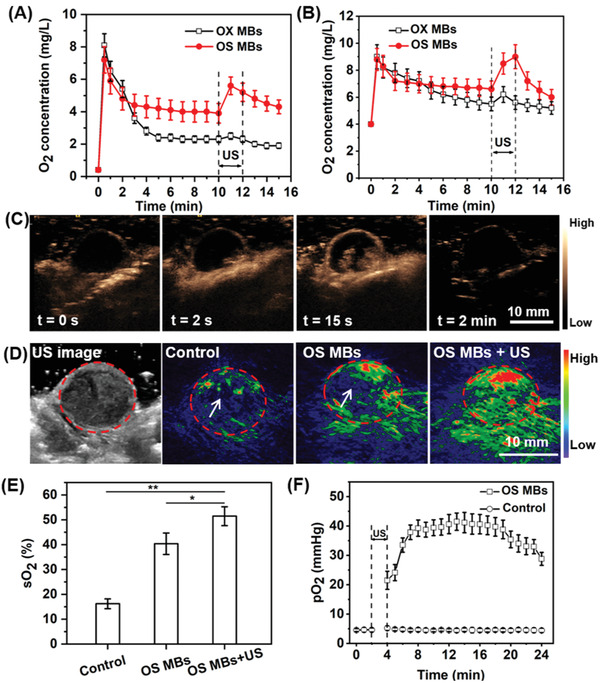
Oxygen release kinetics and in vivo tumor oxygenation of OS MBs. Oxygen release curve of OS MBs and OX MBs with/without two minutes of ultrasound irradiation (MI = 1.2, frequency = 9 MHz) in A) a severely hypoxic solution (oxygen concentration = 0.4 mg L^−1^, *p* = 0.006) and B) a moderately hypoxic solution (oxygen concentration = 4.0 mg L^−1^, *p* = 0.047). C) Perfusion and ultrasound‐triggered destruction of the OS MBs in CNE2 tumors. Ultrasound images of tumors before the administration of the OS MBs (*t* = 0 s), at the initial presence of contrast (*t* = 2 s), at maximum contrast before ultrasound destruction (*t* = 15 s), and after 2 min of ultrasound irradiation (MI = 1.2, frequency = 9 MHz). D) Ultrasound images of tumor region and photoacoustic images (oxyhemoglobin, *λ* = 850 nm) of tumor region in control group (100 µL, PBS), group with the injection of OS MBs (100 µL, ∼2.0 × 10^8^ MBs mL^−1^), and group with the injection of OS MBs followed by 2 min of ultrasound irradiation (MI = 1.2, frequency = 9 MHz, OS MBs + US). The dashed circles indicate the tumor regionand the arrows indicate the relatively hypoxic areas. E) Comparison of oxygen saturation (sO_2_) between the control group, OS MBs group, and OS MBs + US group (*n* = 3, *p = 0.001 and **p = 0.021). F) Measurement of intratumoral pO_2_ variation of CNE2 tumor‐bearing mice for 20 min after the administration of OS MBs and PBS followed by 2 min of ultrasound irradiation (frequency = 9 MHz, MI = 1.2) (*n* = 3, *p* = 2 × 10^−26^).

The oxygen release kinetics of the OS MBs and OX MBs with and without ultrasound exposure under hypoxic conditions were first investigated in vitro, mimicking the hypoxia of solid tumors.^[^ ^9^ ^]^ After injecting the MBs, the oxygen concentration of the hypoxic solution increased significantly within one minute and declined afterward to a relatively steady level (**Figure** **3**A,B). The initial sharp increase in oxygen concentration was mainly caused by the oxygen that dissolved into the solution during the microbubble solution preparation. The oxygen release rate of the OX microbubble solution was higher than that of the OS microbubble solution as a result of the more severe oxygen diffusion across the lipid membrane of the former. Furthermore, the oxygen release of the MBs was slower under moderately hypoxic conditions (oxygen concentration = 4.0 mg L^−1^) than under severely hypoxic conditions (oxygen concentration = 0.4 mg L^−1^). After 10 min, a second increase in the oxygen concentration of the hypoxic solution was observed when exposed to a two‐minute ultrasound burst at an MI of 1.2, which is higher than the inertial cavitation threshold (i.e., MI > 0.8) for MBs typically used as UCAs.^[^ ^32^ ^]^ Accordingly, the OS microbubble solution changed from opaque to clear, which indicated that the OS MBs ruptured to induce oxygen release. Moreover, the second increase observed with the OS MBs was much higher (>1.5×) than that with the OX MBs because more oxygen from the OX MBs had already been released into the hypoxic solution before ultrasound activation due to their poor stability. Owing to the enhanced stability from the presence of SF_6_ in the filling gas, the OS MBs exhibited relatively slow oxygen release under hypoxic conditions, which reduces the risk of premature oxygen release. The entrapped oxygen in the OS MBs could be abruptly released upon ultrasound irradiation.

Next, we performed in vivo ultrasound imaging of tumor‐bearing mice to investigate the biodistribution and ultrasound‐triggered destruction of the OS MBs. As shown in Figure 3C, in contrast to the minimal ultrasound signal within the tumor before injection of the OS MBs (*t* = 0 s), a notable ultrasound signal was initially observed at the boundary of the tumor within approximately 2 s after injection, implying that the OS MBs remained stable without severe bubble coalescence in vivo and passed freely through the pulmonary capillary vessels. Afterward, the ultrasound signal of the OS MBs quickly spread into the interior area of the tumor and reached a peak within ∼15 s, suggesting that the OS MBs could penetrate part of the interior of the tumor tissues via the vasculature network but not the whole tumor region. With ultrasound irradiation at an MI of 1.2 exerted on the tumor area, the ultrasound signal of the OS MBs in the tumor area vanished instantly (Movie S1, Supporting Information), evidencing their inertial cavitation and collapse as a consequence of ultrasound irradiation. After two minutes of ultrasound, the contrast enhancement was no longer present in the ultrasound image of the tumor tissues, showing that all injected OS MBs were fully destroyed. By leveraging the capacity of the OS MBs as UCAs, ultrasound imaging could monitor their transportation in blood circulation and the outcome of oxygen delivery. Moreover, the instant rupture of the OS MBs activated by ultrasound guaranteed the transient release of oxygen, which exposed the hypoxic tumors to an adequate oxygen level for radiotherapy. In contrast, the oxygen release of other oxygen delivery systems (e.g., hyperbaric oxygen therapy, and PFC emulsions) is diffusion‐dependent and cannot afford the transient oxygenation of hypoxic tumors.

To quantify the oxygenation of the tumor tissues, the oxygen saturation (sO_2_) throughout the tumor area with different treatments was determined by measuring the absorbance of oxyhemoglobin (*λ* = 850 nm) and deoxyhemoglobin (*λ* = 750 nm) via photoacoustic (PA) imaging, a well‐established non‐invasive imaging method employed to investigate tumor oxygen dynamics.^[^ ^33^ ^]^ As shown in Figure 3D, a weak PA signal of oxyhemoglobin was present in the peripheral areas of the tumor tissues in the PBS control group, while an enhanced PA signal of oxyhemoglobin was observed in tumor tissues following administration of the OS MBs. With the ultrasound activation, the PA signal of oxyhemoglobin increased even further. More importantly, there was no relatively hypoxic area in any part of the tumor region in the OS MBs + US group, while such areas were observed in the PBS control group and OS MBs groups, implying that ultrasound activation facilitated the release of oxygen to reach deeper hypoxic areas in the tumor tissues. The sO_2_ of the whole tumor regions of different groups showed that administration of the OS MBs in combination with US activation achieved the maximum oxygen delivery efficiency (Figure 3E). To continuously monitor the oxygen level changes in the tumor tissues, pO_2_ at the central position of the tumor area was measured directly. Figure 3F shows that pO_2_ immediately increased to a significant degree (>4.7×) after two minutes of ultrasound activation of the administered OS MBs. The maximum pO_2_ of the OS MB group was approximately eight times higher than that of the control group and remained over 30 mm Hg for about 20 min, where cellular radioresistance is minimal.^[^ ^8^ ^]^ Although the inertial cavitation of MBs might disrupt the blood vessels and blood flow in tumor tissues, the ultrasound‐triggered destruction of MBs filled with octafluoropropane has no impact on tumor oxygenation.^[^ ^34^ ^]^ Therefore, the remarkable enhancement of the oxygen level in the tumors was predominately attributable to the oxygen release of the OS MBs. As demonstrated, ultrasound‐mediated OS MBs could have improved oxygen delivery efficiency via temporally and spatially controlled oxygen release in the tumor region and enhanced oxygen transport within tumor tissues by ultrasound disturbance of the vasculature and extracellular matrix.

### 2.3 Brachytherapy Enhancement under Hypoxic Conditions Using OS MBs

**Figure 4:**
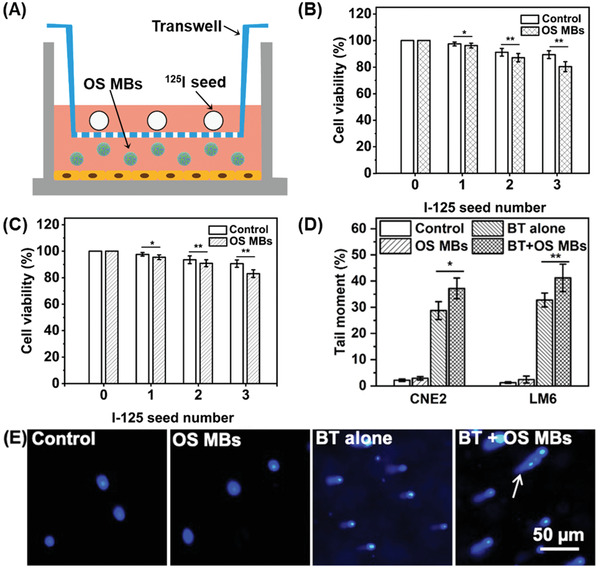
In vitro brachytherapy enhancement of OS MBs for CNE2 and LM6 cells under hypoxic conditions. A) Scheme showing in vitro ^125^I brachytherapy with the addition of OS MBs for oxygenation, where cells were seeded on culture wells at the bottom with ^125^I seeds placed on the transwell and OS MBs were added to the medium. B) Cell viability of CNE2 cells irradiated by the ^125^I seeds at different numbers (∼870 µCi per seed) for 48 h with/without the addition of OS MBs for oxygenation under hypoxic or normoxic conditions (*n* = 3, *p = 0.045 and **p = 0.031). C) Cell viability of LM6 cells irradiated by ^125^I seeds at different numbers (∼870 µCi per seed) for 48 h with/without the addition of OS MBs for oxygenation under hypoxic or normoxic conditions (*n* = 3, *p = 0.041 and **p = 0.030). D) Tail moment of CNE2 and LM6 cells with different treatments: 1) PBS control; 2) addition of OS MBs; 3) brachytherapy alone (BT alone); and 4) brachytherapy and addition of OS MBs (BT + OS MBs) (*n* = 3, *p = 0.038, and **p = 0.015). E) Fluorescent images of CNE2 cells with different treatments after single‐cell gel electrophoresis in a comet assay (the arrow indicates the tail formed by radiation‐induced DNA fragments).

The brachytherapy enhancement of the OS MBs under hypoxic conditions was first evaluated in CNE2 and LM6 cells in a brachytherapy model in vitro (**Figure** **4**A). The OS MBs were added to the culture medium to sensitize the hypoxic cells to radiation. The cell viability of CNE2 and LM6 (Figure 4B,C) showed that addition of the OS MBs greatly increased the brachytherapy efficacy, in contrast to the control group under hypoxic conditions. Furthermore, the enhancement was greater with higher doses, while the cell viability of the PBS group did not greatly change with increasing radiation dose. Since the cells were exposed to hypoxic conditions (1% O_2_), where cellular radioresistance is prominent,^[^ ^8^ ^]^ the outcome of brachytherapy was significantly inhibited in the control group regardless of radiation dose. However, the oxygen release of the OS MBs altered the hypoxic condition and sensitized the cells to radiation to a significant degree. As expected, the addition of the OS MBs did not cause significantly different results from the control group under normoxic conditions, and the normoxia groups showed a similar performance to the hypoxic condition with the addition of the OS MBs. In addition, a comet assay was conducted to evaluate radiation‐induced DNA damage with different treatments.^[^ ^35^ ^]^ As shown in Figure 4E, the radiation‐induced DNA fragments formed a tail (indicated by an arrow) along the nucleus head (bright spots) after single‐cell gel electrophoresis. The tail moment, defined as the product of the percentage of total DNA in the tail and the distance between the centers of the mass of head and tail regions was analyzed across groups with different treatments to reveal the degree of DNA breakage. Figure 4D shows that the addition of the OS MBs caused much more radiation‐induced DNA damage in both CNE2 and LM6 cells by overcoming the hypoxia‐induced cellular radioresistance compared with brachytherapy alone.

**Figure 5:**
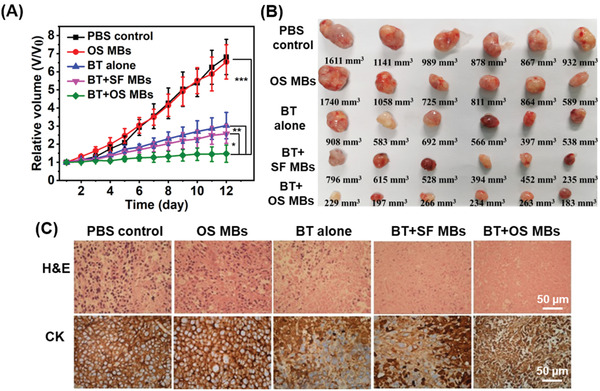
In vivo brachytherapy treatment. The animal treatment protocol was as follows: 1) ^125^I seed implantation; 2) administration of OS MBs, and 3) ultrasound destruction of OS MBs. The mice were categorized into five groups: Group 1, administration of PBS (PBS control); Group 2, administration of OS MBs followed by ultrasound activation (OS MBs); Group 3, brachytherapy alone (BT alone); Group 4: brachytherapy with administration of SF MBs followed by ultrasound activation (BT + SF MBs); and Group 5, ^125^I brachytherapy with administration of OS MBs followed by ultrasound activation (BT + OS MBs). A) Tumor growth curves of different groups with various treatments for twelve days (*n* = 6, *p = 0.001, **p = 0.001, and ***p = 0.0004). B) Representative images of in vivo volume of tumors harvested from euthanized CNE2 xenograft tumor‐bearing nude mice with different treatments. C) Microscopic images of hematoxylin and eosin (H&E)‐ and cytokeratin (CK)‐stained tumor slices collected from mice of different groups after the twelve‐day post‐treatment. For CK staining, CK appears as a brown color stained with 3,3′‐diaminobenzidine, and the nucleus appears as a blue color stained with hematoxylin.

Then, the efficacy of brachytherapy (BT) in conjunction with ultrasound‐mediated oxygen delivery using the OS MBs was assessed in CNE2 tumors in vivo. The mice were divided into five groups: Group 1, PBS control; Group 2, OS MBs; Group 3, BT; Group 4, SF MBs + BT; and Group 5, OS MBs + BT. For BT, the number of implanted seeds for each mouse was first determined using a computerized treatment planning system with a matched peripheral dose of ∼120 Gy (Figure S1, Supporting Information), and ^125^I seeds of the prescribed dose were implanted into the center of the CNE2 tumors (Figure S2, Supporting Information). The tumor growth curves (**Figure** **5**A) revealed that the treatment of Group 3 (BT), Group 4 (SF MBs + BT), and Group 5 (OS MBs + BT) had significant effects (*p* < 0.01) on inhibiting tumor growth compared with the PBS control. Additionally, the results of the PBS control group and Group 2 (OS MBs) demonstrated that the increased oxygen level in the tumor tissues failed to significantly inhibit tumor growth. The effective suppression of tumor growth in Groups 3, 4, and 5 resulted from the local radiation of ^125^I seeds. Compared with BT alone, there were additional therapeutic benefits in both Group 4 (SF MBs + BT) and Group 5 (OS MBs + BT), with Group 5 exhibiting the maximum additional therapeutic benefits. The final tumor volume of each group after different treatments (Figure 5B) also demonstrated that Group 5 had the best therapeutic effect. Furthermore, the tumor tissues in each group were harvested for histological analysis, and thus the survival rates of each group were unavailable. Figure 5C shows that Group 5 (OS MBs + BT) caused the most severe damage to the tumor cells among the groups, and partial tumor cell apoptosis was observed in Group 3 (BT) and Group 4 (SF MBs + BT). Compared with BT alone, the extra tumor control benefits in Group 4 originated from the radiation enhancement induced by the microbubble cavitation effect.^[^ ^36^ ^]^ Microbubble cavitation causes ceramide‐related endothelial cell apoptosis, which leads to vascular disruption and enhances the tumor radiation response.^[^ ^37^ ^]^

Although the tumor radiation response was improved by microbubble cavitation, hypoxia was still the dominant limitation in realizing the full potential of brachytherapy, which was confirmed by the high expression level of HIF‐1*α*, a hypoxia‐related biomarker, as shown in Figure S3, Supporting Information. Compared with Group 4 (SF MBs + BT), the remarkably higher tumor growth inhibition in Group 5 (OS MBs + BT) arose mainly from the successful sensitization of hypoxic tumor cells to radiation by oxygen delivery via the local ultrasound‐triggered destruction of OS MBs,^[^ ^38^ ^]^ which has been observed in PFC nanoemulsion‐based oxygen delivery during external radiotherapy.^[^ ^17^ ^]^ In contrast to the diffusion‐dependent release behavior of PFCs, our OS MBs are acoustic‐responsive with the capacity to instantly relieve tumor hypoxia. The employment of our OS MBs offers significant extra therapeutic gains, which allow for radiation dose reduction, potentially reducing the risk of overdose‐induced side effects (e.g., radiation pneumonitis).^[^ ^39^ ^]^ In addition, no notable acute adverse effects (e.g., pulmonary vascular obstruction) were observed after injection of the OS MBs, indicating that the OS MBs were stable with no severe bubble coalescence or size shift in vivo. Furthermore, the microscopic images of H&E‐stained tissue sections of major organs including the heart, liver, spleen, lung, and kidney demonstrated that the organs were not affected by treatment with the OS MBs (Figure S4, Supporting Information), further confirming the lack of severe side effects caused by their therapeutic administration. To pave the way for further clinical transitions, the OS MBs require more extensive investigations in terms of aspects such as microbubble sterility, effective dosage of MBs, and other types of radiotherapy.

## 3 Conclusion

In this study, we developed a simple emulsion freeze‐drying method to produce MBs filled with a mixture of oxygen and sulfur hexafluoride gases. The OS MBs possess the merits of good stability, easy shelf storage, appropriate size distribution, and high oxygen carrying capacity and are thus well suited for the tumor‐specific, ultrasound‐controlled delivery of oxygen to sensitize hypoxic tumor cells. Due to their excellent ultrasound contrast performance and good stability, the blood transportation of OS MBs and the oxygen delivery outcome can be continuously monitored using ultrasound imaging. Using this oxygen microbubble radiosensitizer, a remarkable improvement in the therapeutic efficacy of ^125^I brachytherapy was demonstrated in CNE2 tumor models, proving that ultrasound‐mediated oxygen delivery with the OS MBs is an effective and robust strategy to realize the full therapeutic potential of brachytherapy for hypoxic tumors.

## 4 Experimental Section

##### Materials

Lipids of 1,2‐distearoyl‐sn‐glycero‐3‐phosphocholine (DSPC) and 1,2‐dipalmitoyl‐sn‐glycero‐3‐phospho‐(1′‐rac‐glycerol) (sodium salt) (DPPG) were purchased from Avanti Polar Lipids (Alabaster, AL, USA). Polyethylene glycol (MW: ∼4000, PEG), mannitol, palmitic acid, and perfluoroheptane were purchased from Sigma‐Aldrich (St. Louis, MO, USA). Sulfur hexafluoride and oxygen were purchased from Jietong Gas Technology (Guangzhou, China). ^125^I seeds (Type 6711, diameter: 0.8 mm, length: 4.5 mm, matched peripheral dose: 100–140 Gy) were purchased from Yunke Pharmaceutical (Chengdu, China). All aqueous solutions were prepared in Milli‐Q deionized water (18 MΩ cm^−1^, Millipore, MilliQ system).

##### Fabrication of MBs

The MBs were prepared by a modified emulsion freeze‐drying method. Briefly, 15 mg of DSPC, 5 mg of DPPG, and 2 mg of palmitic acid were dissolved in 10 mL of a 10% w/v mannitol solution. PFH (1 mL) was added to the lipid solution and emulsified into a microemulsion using a high‐speed homogenizer at 13 000 rpm for 1 min in an ice‐water bath. The PFH emulsion was washed three times with water to remove excess lipids. The resultant PFH emulsion was dispersed in 10 mL of a 10% w/v PEG solution, evenly distributed in glass vials (1.0 mL per vial), and subsequently lyophilized using a freeze‐dryer (FreeZone 4.5, Labconco, USA) for three days. The glass vials containing the lyophilisates were sealed with a rubber septum, and the air in the vials was replaced with filling gas using a home‐built gas exchange system involving a three‐way valve connected to a vacuum line, the sample vial, and the filling gas bottle. Three types of filling gas, including O_2_, mixed gases of O_2_ and SF_6_ at volume ratios of 9:1 to 6:4, and SF_6_ were used to obtain oxygen microbubbles, O_2_/SF_6_ microbubbles, and SF_6_ microbubbles, respectively. For use, microbubble solutions were prepared by suspending the lyophilisates in 4 mL of PBS buffer and diluted with PBS buffer to the desired concentrations.

##### Morphology, Size, and Concentration of MBs

The morphology of the MBs was examined using an inverted epifluorescence microscope (Eclipse Ti‐U, Nikon, Japan). The size and size distribution of the OX, OS, and SF MBs were measured according to the zeta potential (Zeta Plus, Brookhaven Instruments, USA) three times. The concentrations of OX MBs, OS MBs, and SF MBs were measured using a hemocytometer (Neubauer, Germany). For the stability test, 3 mL of OX microbubble, OS microbubble, and SF microbubble solutions (∼2.0 × 10^7^ MBs mL^−1^) were incubated at 37 °C, and the microbubble concentration was measured at specific time intervals (0, 5, 15, 30, and 60 min).

##### In Vitro Oxygen Release of MBs

The oxygen release of the MBs in hypoxic aqueous solutions was measured using a dissolved oxygen meter (JPBJ‐608, Rex, China). A normal saline solution (30 mL) was loaded into a 50 mL centrifuge tube charged with a magnetic stirrer and subsequently sealed with a rubber septum. The centrifuge tube was immersed in a water tank 2 cm from an ultrasound transducer coupled with an ultrasound system (Acuson S2000, Siemens, Germany). The oxygen electrode was inserted into the normal saline solution through a hole punched in the rubber septum, and nitrogen was introduced to the normal saline solution via a long thin needle to obtain hypoxic conditions. When the oxygen concentration reached 0.4 or 4.0 mg L^−1^, the nitrogen bubbling was stopped, and 2 mL of OS MBs (∼2.0 × 10^8^ MBs mL^−1^) was injected into the hypoxic solution. Ten minutes later, the tube was exposed to a 2 min ultrasound burst (MI = 1.2, frequency = 9 MHz) with a pulse duration of 0.43 µs and a pulse repetition frequency of 48 Hz. The output power was ∼2.8 MPa at a distance of 10 mm from the transducer. The oxygen concentration was recorded at specific time points for 15 min. The OX MB solution was tested as a control.

##### In Vitro Brachytherapy

The radiosensitizing effect of the OS MBs was evaluated in CNE2 cells and hepatocellular carcinoma LM6 cells under hypoxic conditions using a ^125^I seed irradiation model.^[^ ^40^ ^]^ Briefly, 500 µL of CNE2 and LM6 cells (∼1 × 10^4^ cells) were seeded in culture wells of a 24‐well plate. The cells were incubated for 8 h in a hypoxic incubator chamber (1% O_2_) or a normoxic incubator (21% O_2_). Then, a transwell containing different numbers of ^125^I seeds (∼870 µCi per seed) was inserted into the culture wells. Twenty microliters of OS MBs (∼2.0 × 10^8^ MBs mL^−1^) was added to the culture medium, and PBS was added as a control. The cells were incubated for 48 h under each condition. An MTT assay was performed to evaluate the cell viability of each group.

##### In Vivo Ultrasound Imaging

To evaluate the biodistribution and ultrasound destruction of the MBs, 100 µL of an OS MB solution (∼2.0 × 10^8^ MBs mL^−1^) was administered to tumor‐bearing mice via a 26 G needle through a tail vein. A transducer 9L4 (4–9 MHz, Siemens, Germany) coupled with an ultrasound system (Acuson S2000, Siemens, Germany) was attached to the tumor with coupling gels for imaging. The ultrasound images were acquired at an MI of 0.1 and a frequency of 9 MHz in cadence pulse sequencing mode. To trigger the MBs, a two‐minute ultrasound burst (MI = 1.2, frequency = 9 MHz) was employed.

##### In Vivo Oxygen Delivery

The oxygen delivery efficiency of the OS MBs was evaluated by monitoring the oxygen saturation within the tumor region via photoacoustic computed tomography (Nexus 128, Endra, Michigan, USA). An OS microbubble solution (100 µL) (∼2.0 × 10^8^ MBs mL^−1^) was injected into CNE2 tumor‐bearing mice via the tail vein (*n* = 3). 2 min of ultrasound irradiation (MI = 1.2, frequency = 9 MHz) was applied to destroy the OS MBs. The treated mice were transferred for photoacoustic imaging with the xenograft tumors fixed in the dimple at the bottom of the bow‐like imaging tray. Optical wavelengths of 750 and 850 nm were selected for deoxyhemoglobin and oxyhemoglobin, respectively.^[^ ^41^ ^]^ Each scan used 120 angles and 60 pulses per angle. The scans were performed with the following parameters: tuning range, 2700–3100 nm; peak energy, 6 mJ; pulse width, 5–7 ns; pulse frequency, 20 Hz; and beam diameter, 4 mm. Each scan cost ∼13.8 min. The photoacoustic images were reconstructed by volumetric rendering using the Osirix software. The PA signal intensity was calculated from the values of the volumetric rendering points. Oxygen saturation (sO_2_) was calculated as (Absorbance of oxyhemoglobin) / (Absorbance of oxyhemoglobin + Absorbance of deoxyhemoglobin) × 100%.

##### In Vivo Therapy

The mice inoculated with CNE2 tumors were randomly distributed into five groups (six mice per group): Group 1, PBS control; Group 2, OS MBs; Group 3, brachytherapy alone; Group 4, brachytherapy + SF MBs; and Group 5, brachytherapy + OS MBs. The animal treatment protocol was as follows: 1) ^125^I seed implantation, 2) administration of OS MBs, and 3) ultrasound destruction of OS MBs. For ^125^I seed implantation, the position of the brachytherapy applicator and the number of implanted seeds for each mouse were determined using a computerized treatment planning system (Unicorn Serence and Technology, Beijing, China) (Figure S1, Supporting Information). The individual tumor model was constructed based on ultrasound images acquired by a portable ultrasonic system (Logic E, GE, USA) before ^125^I seed implantation. The prescribed matched peripheral dose for the mice varied slightly from 120 Gy. ^125^I seeds were implanted into the desired locations of the tumors under ultrasound imaging guidance. For groups with the administration of OS MBs, 100 µL of an OS MB solution (∼2.0 × 10^8^ MBs mL^−1^) was intravenously injected into the mice via a tail vein, followed by 2 min of ultrasound irradiation (MI = 1.2, frequency = 9 MHz) every day. The tumor size was measured by ultrasound imaging every day. After twelve days of treatment, the mice were sacrificed to collect the tumors and major organs, including the heart, liver, spleen, lungs, and kidneys, for histological analysis. The use of animals was approved by the Institutional Animal Care and Use Committee of Sun Yat‐sen University Cancer Center, and the IACUC approval number is L102042018000A.

##### Statistical Analysis

Statistical analysis of all data was performed using OriginPro 2016 (OriginLab, USA) and MS‐Excel 2016(Microsoft, USA). All results are presented as the mean ± standard deviation. A two‐tailed Student's *t*‐test was used to determine the significance between groups. In all cases, *p* < 0.05 was considered statistically significant.

## Conflict of Interest

The authors declare no conflict of interest.

## Supporting information

Supporting Information

Supplemental Movie 1
