# Supplementary material for: A Robust Oxygen Microbubble Radiosensitizer for Iodine‐125 Brachytherapy
Source: Adv Sci (Weinh). 2021 Feb 10;8(7):2002567. doi: 10.1002/advs.202002567 (PMC8025033; doi:10.1002/advs.202002567)
Supplement: Supplementary file 1 — Supporting Information [file ADVS-8-2002567-s002.pdf]

© 2020 Wiley-VCH GmbH

ADVANCED SCIENCE Open Access

Supporting Information

for *Adv. Sci.,* DOI: 10.1002/advs.202002567

A Robust Oxygen Microbubble Radiosensitizer for Iodine-125 Brachytherapy

Sheng Peng, Ruyuan Song, Qingguang Lin, Yanling Zhang, Yuanzhong Yang, Ma Luo, Zhihui Zhong, Xiaonan Xu, Ligong Lu[f], Shuhuai Yao*, Fujun Zhang**

Supplementary Information

A Robust Oxygen Microbubble Radiosensitizer for Iodine-125 Brachytherapy

Sheng Peng[a], Ruyuan Song[b], Qingguang Lin[a], Yanling Zhang[c], Yuanzhong Yang[d], Ma Luo[c], Zhihui Zhong[c], Xiaonan Xu[e], Ligong Lu[f], Shuhuai Yao[b,e]*, Fujun Zhang[c]**

Dr. S. Peng, Dr. Q. Lin
Department of Ultrasound, Sun Yat-Sen University Cancer Center, State Key Laboratory of Oncology in South China, Collaborative Innovation Center for Cancer Medicine, Guangzhou 510060, P.R. China

Dr. R. Song, Prof. S. Yao
Bioengineering Graduate Program, Department of Chemical and Biological Engineering, The Hong Kong University of Science and Technology, Hong Kong, China
Email: meshyao@ust.hk

Dr. Y. Zhang, Dr. M. Luo, Dr. Z. Zhong, Prof. F. Zhang
Department of Imaging and Interventional Radiology, Sun Yat-Sen University Cancer Center, State Key Laboratory of Oncology in South China, Collaborative Innovation Center for Cancer Medicine, Guangzhou 510060, P.R. China
Email: zhangfj@sysucc.org.cn

Dr. Y. Yang
Department of Pathology, Sun Yat-Sen University Cancer Center, State Key Laboratory of Oncology in South China, Collaborative Innovation Center for Cancer Medicine, Guangzhou 510060, P.R. China

Dr. X. Xu, Prof. S. Yao
Department of Mechanical and Aerospace Engineering, The Hong Kong University of Science and Technology, Hong Kong, China.

Prof. L. Lu
Zhuhai Interventional Medical Center, Zhuhai Precision Medical Center, Zhuhai People's Hospital, Zhuhai Hospital of Jinan University, Zhuhai 519000, P.R. China.
Email: lulg@21cn.com

Cytotoxicity of MBs.

The biocompatibility of MBs was evaluated in CNE2 cells. The cells were cultured in RPMI-1640 medium supplemented with 10% fetal bovine serum at 37 °C under 5% CO_2. CNE2 cells (100 µL) (~60,000 cells/ml) were seeded in each well of a 96-well plate and cultured for 24 h. The culture medium was replaced with fresh one containing the OS MBs at different concentrations from 0 to ~3.2×10^9 MBs/mL. The cells were subsequently cultured for 24 h and 48 h, respectively. The cell viability was evaluated by an MTT assay. Twenty milliliters of MTT solution was added to each well and the plate was incubated at 37 °C for another 3 h. After the supernatant of each well removed, 200 µL of DMSO was added. The absorbance value of each well at 570/690 nm was recorded by a plate reader (xMark, Bio-Rad, USA) to evaluate the cell viability at each concentration.

Ultrasound Characterization of MBs

The acoustic property and stability of the MBs were investigated using a diagnostic ultrasound system (Acuson S2000, Siemens, Germany) in a gel phantom, made of a 2% (w/v) agarose solution. The diluted OX MBs, OS MBs and SF MBs (~2.0×10^7 MBs/mL, 1 mL) were loaded into the sample wells in the gel phantom, submerged in the water. Ultrasound images of the MBs in the gel phantom were acquired by the ultrasound system at a frequency of 9 MHz and a mechanical index (MI) of 0.06 at specific time points. The ultrasound images were analyzed by the Image J software to obtain average grey values as the intensity of the ultrasound signal of the MBs.

Comet Assay

The CNE2 cells and LM6 cells were first treated in a ^{125}I seed irradiation model.[1] Briefly, 500 μL of CNE2 and LM6 cells (~1.0 × 10^4 cells) were seeded in the wells of a 24-well plate respectively and divided into four groups: Group 1, Control; Group 2, OS MBs; Group 3, BT alone; and Group 4, BT + OS MBs. For groups with the addition of OS MBs, 20 μL of the OS microbubble solution (~2.0 × 10^8 MBs/mL) was added into the culture medium and PBS was added for the control group. As for groups with BT, a transwell containing the ^{125}I seeds (~2,600 μCi) was inserted into the culture well. All groups were incubated in a hypoxia incubator chamber (1% O_2) for 48 h. The cells were collected and suspended in PBS at a concentration of ~1.0 × 10^6 cells/mL.

An comet assay was performed to analyze the radiation-induced DNA damage of cells with different treatments, following a protocol.[2] A 0.8% agarose solution (100 μL) at 60 ℃ was dropped on a microscope glass slide and a cover slip was placed on the top of the gel. After the gelation and the removal of the cover slip, the freshly prepared mixture of the cell suspension (10 μL) and a 1.0% low-gelling-temperature agarose solution (100 μL) was coated on the top of the gel immediately. During the gel fabrication, bubbles should be avoided all the time. After the fixation of the cells in the gel, the glass slide was immersed in a lysis buffer (2% sarkosyl, 0.5 M EDTA, 0.5 mg/mL proteinase K, pH 8.0) for one hour at 4 ℃. Then, the glass slide was incubated in an alkaline buffer (0.03 M NaOH, 2 mM EDTA, pH 12.3) for 30 min for the DNA unwinding. The single-cell electrophoresis of the glass slide was performed at a voltage of 25 V and a current of 300 mA for 15 min. The glass slide was washed with 0.4 M Tris-HCl buffer (pH 7.5) and 75% ethanol subsequently. The glass slide was stained with 5 μg/mL DAPI solution for 20 min and examined by a fluorescence microscope (DM250, Leica, Germany). The tail moment, defined as the product of the percentage of total DNA in the tail and the distance

between the centers of the mass of head and tail regions, of each group was analyzed based on the fluorescent images. [Tail moment = (tail mean-head mean) × % of DNA in the tail]

Animal Model

Female BALB/c Nude mice in the study were purchased from Vital River Laboratories (Beijing, China) and treated in accordance with the protocol approved by The Institutional Animal Care and Use Committee of Sun Yat-sen University Cancer Center and the IACUC approval number is L102042018000A. The mice were anesthetized using pentobarbital and 50 μL of PBS containing CNE2 cells (~1.0×10^6 cells/mL) was subcutaneously injected into the hind leg of each mouse. After two weeks, the volume of the xenograft tumors reached ~200 mm^3. The tumor size was measured by the ultrasound imaging using a portable ultrasonic system (Logic E, GE, USA) and the tumor volume was calculated using the modified ellipsoid formula (i.e., $0.5 \times (\text{Length} \times \text{Width}^2)$) based on the ultrasound images.

Intratumroal Oxygen Level Measurement

The intratumoral oxygen level at the center of the tumor was directly measured using a microelectrode needle (OX-25, Unisense, Denmark) (outside tip diameter: 20~30 μm, total length: 150~200 mm, oxygen consumption rate: 4.0×10^{-4}~5×10^{-3} nmol/h), connected to an oximeter (Microsensor Multimeter, Unisense, Denmark). An ultrasound transducer 9L4 (4~9 MHz, Siemens, Germany) was attached on the tumor tissue with the coupling gel and the microelectrode needle was placed in the center of the tumor tissue under the guidance of ultrasound imaging. One hundred microliters of the OS microbubble solution (~2.0×10^8 MBs/mL) was injected into the CNE2 tumor-bearing mice. Afterwards, an ultrasound irradiation (MI = 1.2 and frequency = 9 MHz) was applied to destroy the administrated OS MBs. The

intratumoral pO_2 was recorded at the specific time intervals for twenty minutes. The PBS was used as the control.

Histological Analysis

After the *in vivo* brachytherapy, three tumors from each group were dissected to make frozen formalin-fixed sections, stained with hematoxylin and eosin (H&E) and antibodies specific for cytokeratin AE1/AE3, respectively, in order to assess the apoptosis and necrosis of the tumors with different treatments. Furthermore, the excised major organs including the heart, liver, spleen, lung, and kidney were stained with H&E to evaluate the safety of the OS MBs. For the hypoxia-related biomarker HIF-1α analysis, three tumors from mice before/after the treatment of the brachytherapy in combination of OS MBs were sampled and stained with anti-HIF-1α antibody to assess the expression level of HIF-1α. Finally, the excised tissue slices were examined with a fluorescence microscope (DM250, Leica, Germany) for the histological analysis.

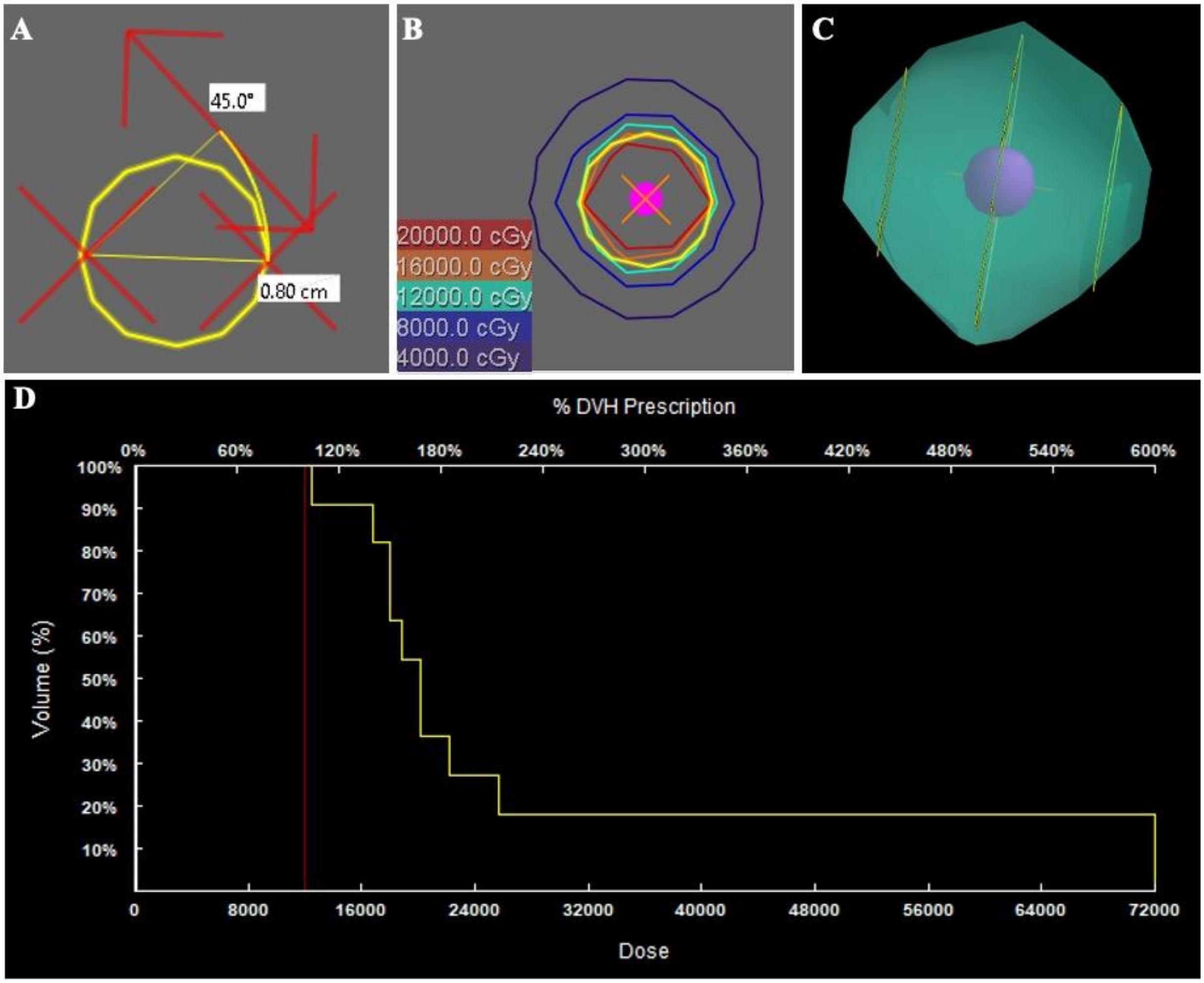

Figure S1. A case of treatment planning system (TPS) for tumor model with a diameter of 0.80 cm. **(A)** Two-dimensional image describes the targeted tumor area for the TPS calculation. **(B)** Two-dimensional image shows the seeds (purple), the tumor's contour (yellow), and the dose contour (indicated by different colors). **(C)** Three-dimensional image shows the seeds (purple), tumor area (green), and the dose contour (12,000 cGy). **(D)** Dose–volume histogram (DVH) shows the prescribed dose to the tumors (reference dose: 12,000 cGy, indicated by red-color line), calculated by the TPS.

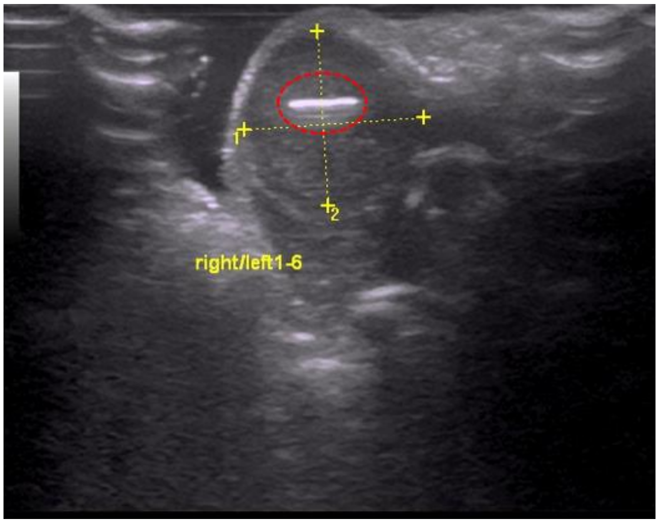

Figure S2. Ultrasound image of the CNE2 tumor with ^{125}I seeds implanted. The bright bar in the red circle indicates the seeds.

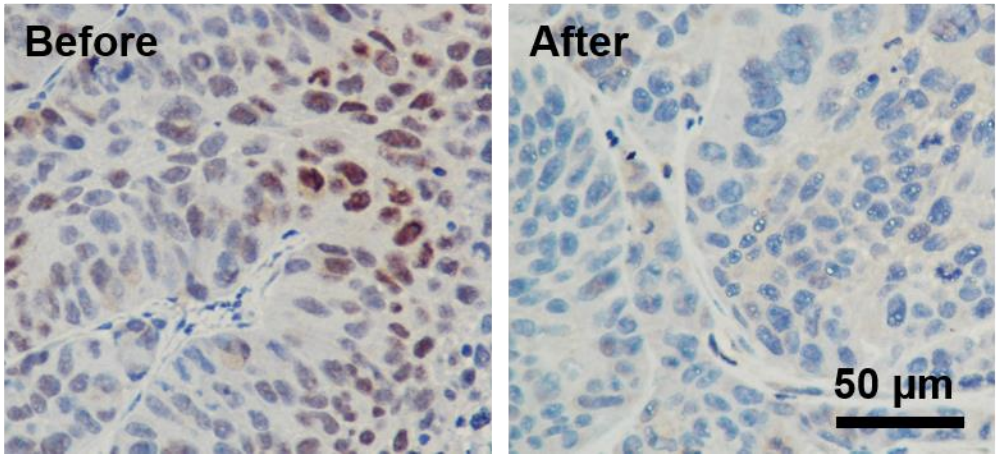

Figure S3. Microscopy images of the HIF-1α stained tumor slices collected from the mice before/after the twelve days of the treatment with the brachytherapy in combination of OS MBs. The HIF-1α appears as a brown color stained with the 3,3′-diaminobenzidine and the nucleus appears as a blue color stained with the hematoxylin and eosin.

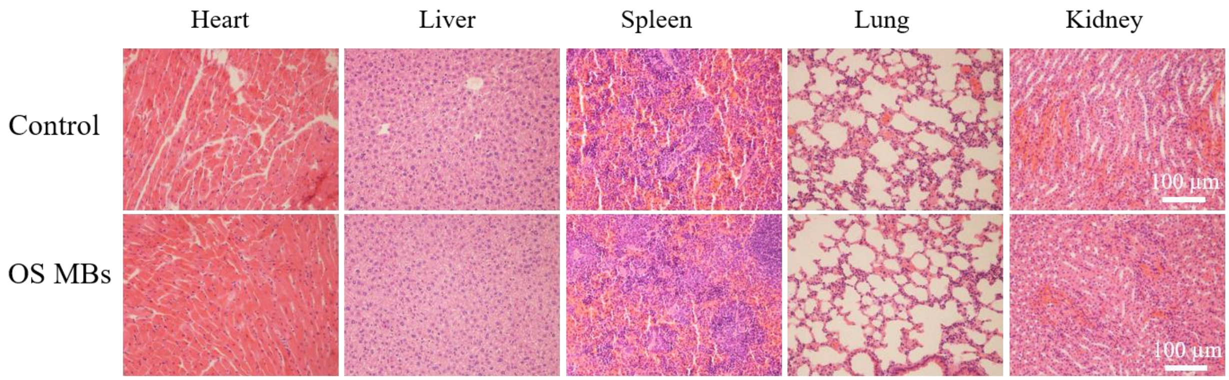

Figure S4. Hematoxylin and eosin stained tissue sections of the major organs including heart, liver, spleen, lung, and kidney of the healthy mice and mice treated with the brachytherapy in combination of OS MBs for twelve days.

References

[1] G. Yang, S. Peng, Y. Zhang, Z. Liu, M. Lu, T. Zhang, F. Gao, F. Zhang, *Recent Pat. Anticancer Drug Discov.* **2014**, 9, 258.

[2] P. L. Olive, J. P. Banáth, *Nat. Protoc.* **2006**, 1, 23.
